# A novel channel selection method for multiple motion classification using high-density electromyography

**DOI:** 10.1186/1475-925X-13-102

**Published:** 2014-07-25

**Authors:** Yanjuan Geng, Xiufeng Zhang, Yuan-Ting Zhang, Guanglin Li

**Affiliations:** 1 Key Laboratory of Human-Machine-Intelligence Synergic System of Chinese Academy of Sciences (CAS), Shenzhen Institutes of Advanced Technology (SIAT), CAS, Shenzhen, China; 2 Key Laboratory of Health Informatics of CAS, SIAT, CAS, Shenzhen, China; 3 National Research Center for Rehabilitation Technical Aids, Beijing, China; 4 Department of Biomedical Engineering of the Chinese University of Hong Kong, Hong Kong, China; 5 Institute of Biomedical and Health Engineering, Shenzhen Institutes of Advanced Technology, University Town of Shenzhen, CAS, 1068 Xueyuan Blvd., Shenzhen, China

**Keywords:** Myoelectric control, Channel selection method, Multi-class common spatial pattern, High-density EMG, Pattern recognition

## Abstract

**Background:**

Selecting an appropriate number of surface electromyography (EMG) channels with desired classification performance and determining the optimal placement of EMG electrodes would be necessary and important in practical myoelectric control. In previous studies, several methods such as sequential forward selection (SFS) and Fisher-Markov selector (FMS) have been used to select the appropriate number of EMG channels for a control system. These exiting methods are dependent on either EMG features and/or classification algorithms, which means that when using different channel features or classification algorithm, the selected channels would be changed. In this study, a new method named multi-class common spatial pattern (MCCSP) was proposed for EMG selection in EMG pattern-recognition-based movement classification. Since MCCSP is independent on specific EMG features and classification algorithms, it would be more convenient for channel selection in developing an EMG control system than the exiting methods.

**Methods:**

The performance of the proposed MCCSP method in selecting some optimal EMG channels (designated as a subset) was assessed with high-density EMG recordings from twelve mildly-impaired traumatic brain injury (TBI) patients. With the MCCSP method, a subset of EMG channels was selected and then used for motion classification with pattern recognition technique. In order to justify the performance of the MCCSP method against different electrode configurations, features and classification algorithms, two electrode configurations (unipolar and bipolar) as well as two EMG feature sets and two types of pattern recognition classifiers were considered in the study, respectively. And the performance of the proposed MCCSP method was compared with that of two exiting channel selection methods (SFS and FMS) in EMG control system.

**Results:**

The results showed that in comparison with the previously used SFS and FMS methods, the newly proposed MCCSP method had better motion classification performance. Moreover, a fixed combination of the selected EMG channels was obtained when using MCCSP.

**Conclusions:**

The proposed MCCSP method would be a practicable means in channel selection and would facilitate the design of practical myoelectric control systems in the active rehabilitation of mildly-impaired TBI patients and in other rehabilitation applications such as the multifunctional myoelectric prostheses for limb amputees.

## Introduction

Surface electromyography (EMG) is an electrical manifestation of muscle contractions, which has been used as a convenient and proprioceptive control signals for the operations of limb prostheses [1], assistive exoskeletons [2], and rehabilitation robots [3]. In recent years, one of the most popular applications of EMG signals would be to use EMG-based pattern recognition (EMG-PR) algorithms to control multifunctional powered prostheses [1,4-7]. More recently, the EMG-PR also was adopted in the neural rehabilitation of the motor impaired patients who suffer from neurological injuries for development of an active rehabilitation system. The previous studies have proved that the active rehabilitation is promising in enhancing the therapeutic effect [8] and accelerating the brain plasticity [9]. In light of these findings, the EMG-PR algorithm was proposed to identify the motor intentions of stroke survivors and patients with incomplete cervical spinal cord injury [10,11]. The results of these previous studies have suggested that EMG-PR algorithms would be feasible and useful in active rehabilitation of patients suffering from neurological injuries.

High density EMG recordings have been increasingly applied in EMG-PR algorithms [12-14] with an attempt to capture more temporal and spatial information about the muscle activities and electrophysiology. With more channels of temporal information that can reflect the activities of some small forearm muscles, a better motion classification performance could be achieved when using high density EMG recordings [13,14]. And the topographical maps of EMG amplitude can be used to examine the exact electrode locations where the strong myoelectric activity is experienced during a motion task [12-14]. However, the data processing of high-density EMG recordings from a large number of EMG electrodes is computationally expensive, making it impractical in real-time myoelectric control. Additionally, the high dimensional EMG recordings may cause the classifier to over-fit the training data due to the irrelevant or redundant information. Therefore, selecting an appropriate number of EMG channels with desired classification performance and determining the optimal placement locations of electrodes would be necessary in the practical myoelectric control for a potential user.

Currently, there are two commonly used ways to determine the number and the locations of EMG electrodes in the applications of EMG-based motion classification. One direct way is based on the physiologically known anatomical knowledge of skeletal muscles [15-17], in which the electrode sites would be chosen by viewing the intensity, repeatability and consistency of multi-channel EMG signal recordings. For the persons with the intact functions of skeletal muscles, using the muscle-physiology-based way we may quickly and easily determine the appropriate sites of EMG electrodes since their almost consistent muscle anatomy could ensure producing very similar muscle contractions when they actuate a limb activity. However, for the persons with some muscular issues such as the patients with a post-stroke, traumatic brain injury or limb amputation, when doing a limb activity, their muscle contractions would be different from healthy people and may be various between patients since the extents, the positions and the causes of their muscular damages might be inconsistent. Thus it would be hardly to use this kind of clinical method for determining the appropriate electrode sites in patients.

Another way to determine a subset of appropriate EMG channels is based on a certain optimizing criterion. A commonly used method is the sequential forward selection (SFS)-based channel selection algorithm [14,16], in which the most informative EMG channels were determined by an optimization procedure with an optimizing objective of high motion classification accuracy. Since SFS method requires to repeatedly perform the searching procedure for the optimal channels until obtaining a given number of channels, this classification-accuracy-based method would take a long computation time to get a relatively optimal subset of EMG channels. Moreover, SFS method must rely on a specific set of EMG features and a specific EMG pattern-recognition algorithm to do the selection of the optimal channels for an EMG control application. When the EMG features and/or pattern-recognition algorithm would be changed for some reasons such as trying to use different features or algorithms for higher classification performance, the selected EMG channels might be not optimal yet. Thus the optimization procedure would be executed again to search the optimal channels accordingly. Some feature-dependent algorithms such as the quasi-optimal channel selection method based on partial Kullback-Leibler information [18] and coefficient-based channel selection method using principal component analysis [19] also have been proposed and used for channel selection. In addition, another two feature selection methods, Fisher-Markov selector (FMS) based on Fisher criterion [20] and the minimal-redundancy-maximal-relevance based on mutual information [21], also fall into the group of feature-dependent channel selection methods. It is obvious that these feature-dependent channel selection methods would rely on the EMG features. Similarly, when changing the set of EMG features such as increasing/decreasing a feature to/from it, the selected channels might not work well again, thus another channel selection procedure would be required to re-determine the appropriate channels accordingly.

Alternatively, the direct channel selection method (i.e., variable selection) which directly works on the raw EMG data also has been proposed to determine a set of appropriate channels for EMG recordings. The Monte Carlo method reported by Nagata et al. for hand motion classification is one of such kinds of the direct channel selection approaches [22]. Unlike the classification-accuracy-based and the feature-dependent channel selection methods, this raw-data-based method is independent of EMG features and classification algorithms. Thus a unique set of optimal EMG channels might be determined from the multi-channel EMG recordings. As a result, when the features and/or classification algorithm are changed, the selected EMG channels would be retained. In this study, we proposed a novel direct channel selection approach named multi-class common spatial pattern (MCCSP) for channel selection in EMG pattern-recognition-based movement classification. Since MCCSP would be independent on EMG features and classification algorithms, it would be more convenient for channel selection in developing an EMG control system than the exiting methods. The performance of the proposed MCCSP method in selecting an optimal set of EMG channels was assessed with high-density EMG recordings from twelve mildly-impaired traumatic brain injured (TBI) patients. Generally, the TBI patients would suffer from the physical and behavioral disorders, so appropriately physical therapy should be employed for the rehabilitation of their physical functions. The EMG-control active rehabilitation systems would be a promising way for TBI patients’ neural function recovery [23]. In addition, the performance of the proposed MCCSP method was compared with that of two exiting channel selection methods, SFS and FMS.

## Methods

### Data collection

**Figure 1:**
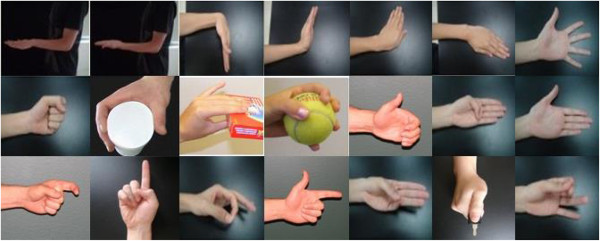
21 classes of forearm and hand movements included in the study.

Twelve mildly-impaired male patients with TBI participated in this study. They were chosen based on the upper limb motion impairment level assessed by a physical therapist. According to the definition of the stages in the Brunnstrom Assessment Scale, they were in stage IV-V and got the scores of 49 to 61 with the Fugl-Meyer Assessment of Sensorimotor Recovery after stroke, in which a zero score denotes no any function and a score of 66 designates as normal function. All of the subjects did not have any experience of attending this kind of research study before. In the experiment, they were asked to use their unilateral arm with severer motor impairment to perform 21 forearm and hand movements (Figure 1) plus one “no movement”. Each movement was maintained for 6 s with a moderate force and repeated 6 times. A rest time of 8 s was set between two successive movements in each trial. All subjects could choose to finish all or part of the 22 movements based on their own motor ability. The Research Ethics Board of the Shenzhen Institutes of Advanced Technology, Chinese Academy of Sciences, approved the experimental protocol of this study, and each subject gave written informed consent and provided permission for publication of photographs for a scientific and educational purpose.

**Figure 2:**
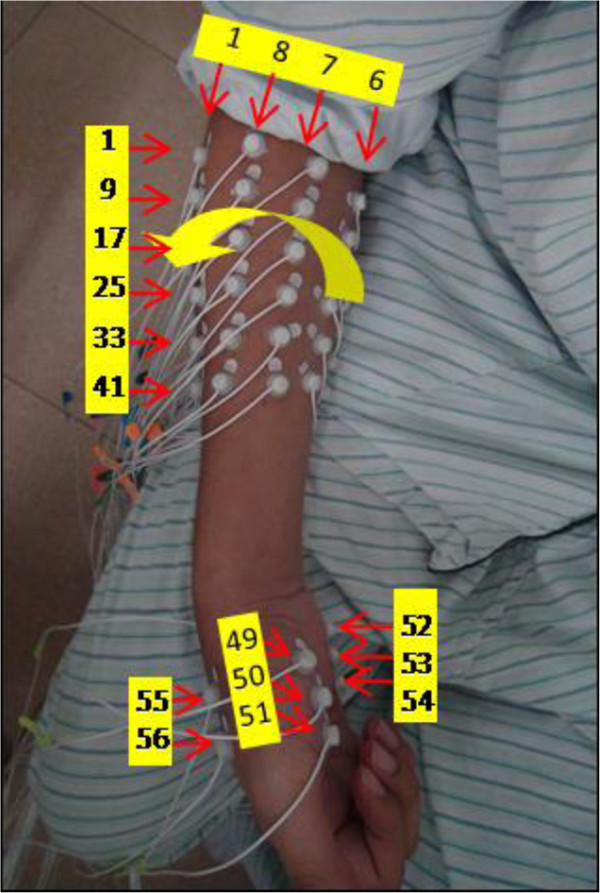
Electrode placement in the experiments.

The high-density EMG acquisition system (*Refa-128, TMS International BV, Netherlands*) was used to record the EMG signals during the experiment. The 56 monopolar electrodes (5 mm in diameter) were placed on the forearm and hand of subjects, as shown in Figure 2. The 48 of 56 electrodes were placed on the forearm in an 8 × 6 grid from 1 cm proximal to the elbow crease to 1/3 distal to the wrist joint with an electrode inter-distance of around 2 cm and other eight electrodes were placed on the hand muscles with two electrodes on the first dorsal interosseous, three on the thenar group muscles, and three on the hypothenar group muscles. A reference electrode was fixed on a nylon bracelet that was worn on subject’s wrist. The sampling rate of EMG signals was set as 1024 Hz.

### EMG data preprocessing

**Figure 3:**
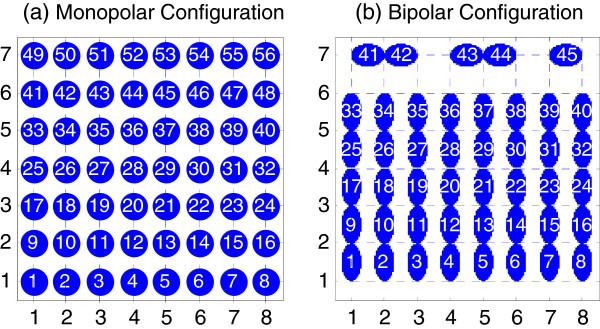
**Two electrode configurations. (a)** 56-channel monopolar electrode configuration **(b)** 45-channel bipolar electrode configuration.

The EMG signals recorded with 56 electrodes were digitally filtered with a five-order Butterworth high-pass filter at 30 Hz, and then the 50 Hz power line interference was reduced with a notch filter from EMG recordings. Two electrode configurations, monopolar mode and bipolar mode, were considered in the study with an attempt to see if the two modes of EMG signals would provide different performance in classifying the movements. The raw recordings were 56 monopolar EMG signals (Figure 3(a)). The bipolar EMG recordings were formed with the differential values between two adjacent monopolar channels along the orientation of muscle fibers from the 56 monopolar EMG signals, resulting in 45-channel bipolar EMG signals, as shown in Figure 3(b).

### EMG channel selection

The proposed MCCSP-based channel selection algorithm as well as the SFS and FMS were applied on the two modes of high-density EMG recordings to select an optimal set of EMG channels (1-20), respectively.

#### MCCSP algorithm

MCCSP-based channel selection algorithm considers the EMG data from all the channels simultaneously. To clearly explain its principle and implementation, the principle of two-class common spatial pattern (CSP) algorithm, the CSP-based channel selection algorithm, and the MCCSP-based channel selection algorithm were successively introduced as follows.

#### CSP algorithm

CSP is a supervised two-class filter algorithm by calculating the linear spatial filters that maximize the variance of one class and meanwhile minimize the variance of another class [24,25]. In this way, the two classes can be maximally separated by their variances. Suppose we want to classify two motion tasks, class A and class B, and use *X*_*A*_ and *X*_*B*_ to denote their corresponding signal matrix with a dimension *n* × *d*, where *d* is the number of channels and *n* is the number of samples per channel. The object becomes to find a spatial filter matrix *W* which can maximize the variance of class A and minimize the variance of class B, which would correspond to an optimization problem that can be formulated as:

(1) $$W=\underset{W}{\arg\max}\frac{W^{T}\Sigma_{A}W}{W^{T}\Sigma_{B}W}$$

By solving a generalized eigenvalue problem, the linear spatial filter matrix *W* can be obtained by simultaneously diagonalizing the covariance matrix Σ_*A*_ and Σ_*B*_:

(2) $$W\Sigma_{A}W^{T}=D_{A}\;W\Sigma_{B}W^{T}=D_{B}\;D_{A}+D_{B}=I$$

where $\Sigma_{A}=1/\left(n-1\right)*X_{A}*X_{A}^{T}$ and $\Sigma_{B}=1/\left(n-1\right)*X_{B}*X_{B}^{T}$. With the constraint condition *D*_*A*_ + *D*_*B*_ = *I*, the eigenvectors with the largest eigenvalues for *D*_*A*_ have the smallest eigenvalues for *D*_*B*_ and vice versa. Applying the filter matrix *W* to the raw signals *X* would give *d* output signals *Y* = *W* * *X* that were also called components. The variance of each component is indicated by its corresponding eigenvalue on the principal diagonal of *D*_*A*_, for class B of *D*_*B*_. In the binary classification problem of EEG signals, the first component that has the largest variance for class A and the smallest variance for class B and the last component that has the smallest variance for class A and the largest variance for class B were commonly used [25].

#### CSP-based channel selection

With *W*^-1^ (inverse matrix of *W*) that is called spatial patterns, the original signal matrix *X* can be reconstructed by *X* = *W*^-1^ * *Y*. The first and last columns of *W*^-1^ would be the most important spatial patterns that explain the largest variance of one motion and the smallest variance of the other. If considering the coefficients of spatial patterns as projection weights, the two channels corresponding to the maximal coefficients of spatial pattern vectors may be the channels that are most correlated with the motion specific sources. This method has been used by Wang et al. to select four EEG channels from 118 channels for classifying three motor imaginary tasks and achieved a high classification accuracy [24].

#### MCCSP-based channel selection

Given a problem with multiple classes of movements in the study, we firstly extended the two-class CSP into multi-class form by using one-versus-rest (OvR) scheme, as done in [26]. For each motion class, a corresponding filter matrix *W*_*C*_^-1^ was calculated by maximizing the variance of the class and minimizing the sum of the variances of all other classes:

(3) $$W_{C}=\underset{\mathrm{Wc}}{\arg\max}\frac{W_{C}^{T}\Sigma_{C}W_{C}}{W_{C}^{T}\left(\Sigma_{i\neq C}\Sigma_{i}\right)W_{C}}$$

Accordingly, multiple spatial pattern matrices corresponding to all the included motion classes would be obtained, where c denotes the class label. Then we used these spatial pattern matrices to select the optimal channels for subsequent pattern recognition analysis. In the study, we adopted similar coefficients-based channel selection method as did in the two-class CSP. For each spatial pattern matrix corresponding to a specific motion class, the two channels corresponding to the maximal coefficients of the first and the last spatial pattern vectors were selected, and then all the selected two-channel from each spatial pattern matrix *W*_*C*_^-1^ were combined together, which were then used as the optimal channels in the subsequent pattern recognition analysis. Note that for the two channels selected from a specific spatial pattern matrix, e.g., *W*_*A*_^-1^, one or both of which may be overlapped with that obtained from another spatial pattern matrix *W*_*B*_^-1^. So the selected channels for all movements were ranked in the order of their occurrence frequency from high to low. The number of selected EMG channels might vary for different subjects.

#### SFS algorithm

SFS is an iterative searching procedure, in which one optimal channel that produces the highest classification accuracy was firstly selected among all the channels, and then another channel that can achieve the maximum classification accuracy with the combination with the selected channels was added [14,16]. Suppose we require to choose an optimal subset with *n* channels from all the 56 channels, the classification computation procedure would be repeatedly implemented by (56 + 56 + 1 - *n*) * *n*/2 times.

#### FMS algorithm

FMS is essentially a feature selection algorithm, which was proposed to select the globally optimal subset of features from high-dimensional feature space in the spirit of Fisher’s class separation criterion [20]. By using specific kernel functions and the Markov random field optimization techniques, the FMS is capable of selecting features and has efficient computational complexity. In this study, we firstly applied FMS to the EMG feature matrix to get the indices of all the features that were ranked in the order of their importance coefficients, and then the feature indexes were mapped to their corresponding channel indexes. In this way, the optimal EMG channels could be selected.

### Feature extraction and motion classification

With a selected subset of EMG signals, a shifting analysis window with a time length of 150 ms and an increment of 100 ms (50 ms overlapping) was used to segment the EMG signals into a series of analysis windows. Then two commonly used time-domain feature sets were extracted from each analysis window, respectively. They were (1) four time-domain features (TD) [6], mean absolute value (MAV), number of zeros crossings (ZC), number of slope sign changes (SSC), and waveform length (WL), and (2) the six order autoregressive (AR) model coefficients [27] plus the root mean square (RMS) amplitude of EMG signals (TDAR). For each analysis window, the features extracted from all the selected EMG channels was concatenated to form a feature matrix, which was then fed into a classifier for motion classification.

Two classifier algorithms, the linear discriminant analysis (LDA) and the k-nearest neighbor (KNN), were used in this study. The LDA classifier was used for its merit of algorithm simplicity and low computational cost [5], and the KNN classifier was used for its tolerance to arbitrary data distribution [28]. In order to find the best feature-classifier combination for each selected EMG subset, each of the two classifiers was separately combined with each of the two feature sets, yielding four different feature-classifier combinations.

### Performance evaluation

The classification accuracy, defined as the percentage of the number of correct classification decisions over the total number of classification decisions, was used to assess the motion classification performance [7]. Five-fold cross validation was performed in motion classification when using the EMG subset selected via MCCSP and FMS for the purpose of evaluating their performance in EMG-PR. The EMG feature matrix extracted from the selected EMG recordings was randomly divided into five subsets with equal length. Four of the five subsets were used as a training set and the remaining one subset was considered as a testing set in each classification process to compute the classification accuracy. The classification accuracies were averaged over the five validation results from the folds. For the EMG subset selected by SFS method, the first half of the feature matrix was used as the training set and its second half was used as the testing set. No cross validation was conducted herein considering the newly added EMG channel was determined according to the maximum classification accuracy during last round repetition.

### Statistical analysis

To assess the statistical difference among the three channel selection methods, one-way ANOVA was performed in terms of motion classification accuracy with SPSS Statistical Modeling Software (SPSS 17.0 IBM Corp., Chicago, IL). In addition, we also analyzed the effect of feature set, classifier, and combination of feature set and classifier on the classification performance by performing two-way ANOVA. The interaction effect of feature set and classifier on classification performance was firstly assessed. If the effect was insignificant, the main effects of features set and classifiers were examined. The level of statistical significance was set to p < 0.05.

## Results

### Number of selected EMG channels using MCCSP

**Figure 4:**
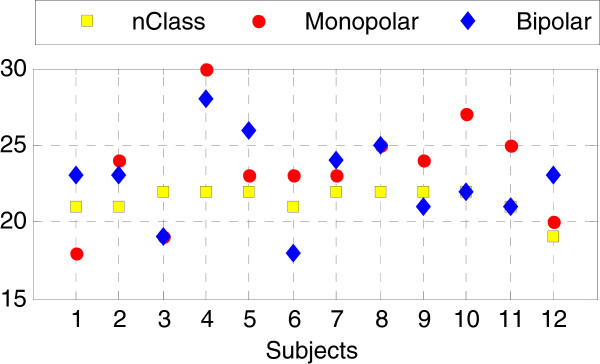
**Number of selected EMG channels when using MCCSP.** The red dots, blue diamonds, and yellow squares denote the number of selected monopolar EMG channels, the number of bipolar EMG channels, and the number of movement classes (nClass) completed by a subject, respectively.

Figure 4 illustrates the numbers of selected EMG channels by MCCSP for all the 12 subjects when using EMG signals from monopolar and bipolar channel configurations, respectively. It can clearly observe from Figure 4 that the number of selected EMG channels varied for different subjects, ranging from 18 to 30 for the two channel configurations. And we can see that the numbers of selected channels by using monololar and bipolar EMG signals, respectively, were similar (in 10 subjects) or same (in two subjects). In order to make the following results comparable among all the subjects, the maximum number of selected EMG channels was set as 18. Note that for a subject the number of selected channels were corresponding to the movement classes that could be completed by him/her. Since the motor impairment of all the subjects was different, the number of completed movement classes (*nClass* designated by the yellow squares in Figure 4) would vary with a range of *nClass* = 19-22.

### Optimal feature-classifier combination

**Figure 5:**
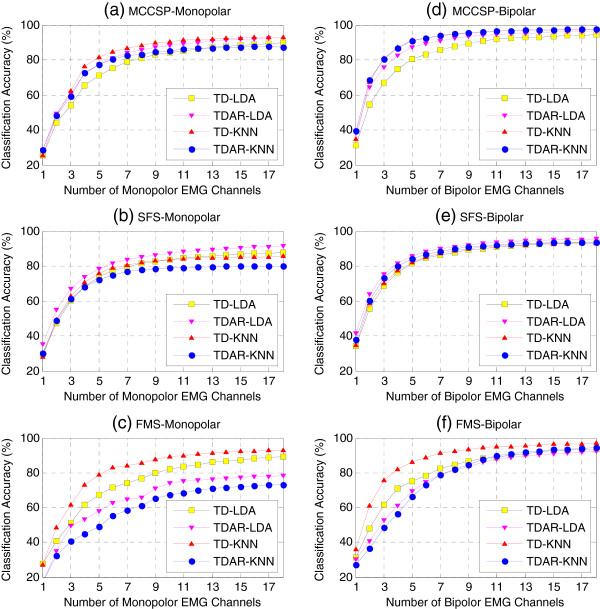
**Comparison of feature-classifier combinations in terms of classification performance.** The average classification accuracy across all subjects was calculated when using four different feature-classifier combinations and three different EMG subsets selected via MCCSP **(a)(d)**, SFS **(b)(e)** and FMS **(c)(f)**, respectively. Both monpolar electrode configuration **(a)(b)(c)** and bipolar electrode configuration **(d)(e)(f)** were considered.

**Figure 6:**
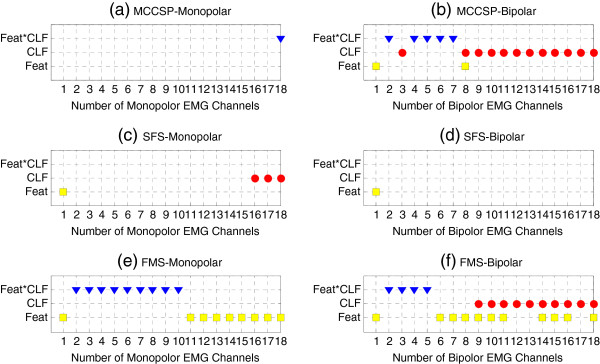
**Impact of feature set, classifier, and feature-classifier combination on classification performance.** The selected EMG subset was determined by using MCCSP **(a)(b)**, SFS **(c)(d)**, and FMS **(e)(f)** method, respectively. Both monopolar electrode configuration **(a)(c)(e)** and bipolar electrode configuration **(b)(d)(f)** were considered. The blue triangles denote significant interaction effect of feature set and classifier, the red dots and yellow blocks denote significant main effect of classifier and feature set, respectively.

To find the optimal feature-classifier combination for each selected EMG subset, the average classification accuracy across all subjects were calculated and shown in Figure 5 when using TD-LDA, TDAR-LDA, TD-KNN, and TDAR-KNN, respectively. The x-axis denoted the number of involved EMG channels that were selected by using MCCSP (Figure 5(a) and (d)), SFS (Figure 5(b) and (e)), and FMS (Figure 5(c) and (f)), respectively. These results show that with monopolar configuration (Figure 5(a)-(c)), TD-KNN outperformed other three feature-classifier combinations when using the EMG subsets determined by MCCSP and FMS, while TDAR-LDA was the best feature-classifier combination when using the EMG subset selected by SFS. It was almost the same case for bipolar configuration (Figure 5(d)-(f)). The difference was that when using the EMG subset selected by MCCSP, TDAR-KNN and TD-KNN had almost the same classification performance. In addition, when using the EMG subset determined by SFS, the difference among all the four feature-classifier combinations became smaller.We further investigated whether the feature set, classifier, or the combination of feature and classifier had a significant impact on the classification performance when using the EMG subset determined by MCCSP (Figure 6(a) and (b)), SFS (Figure 6(c) and (d)), and FMS (Figure 6(e) and (f)), respectively. The results demonstrate that when using the monopolar EMG channels determined by MCCSP (Figure 6(a)), the monopolar EMG channels determined by SFS (Figure 6(c)), and the bipolar EMG channels determined by SFS (Figure 6(d)), the classification performance was relatively stable with respect to different feature sets and classifiers. However, when using the bipolar EMG channels selected by MCCSP (Figure 6(b)), the monopolar EMG channels selected by FMS (Figure 6(e)), and the bipolar EMG channels selected by FMS (Figure 6(f)), the classification performance became sensitive to the choice of feature set and/or classifier.

### Comparison of channel selection methods and electrode configuration

**Figure 7:**
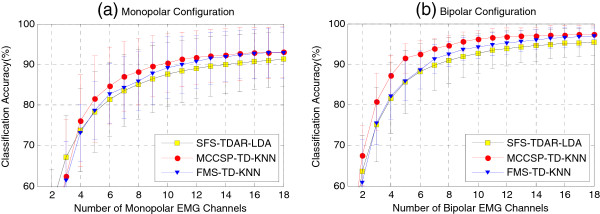
**Comparison of channel selection methods in terms of classification accuracy.** Both **(a)** monopolar electrode configuration and **(b)** bipolar electrode configuration were used, respectively.

**Figure 8:**
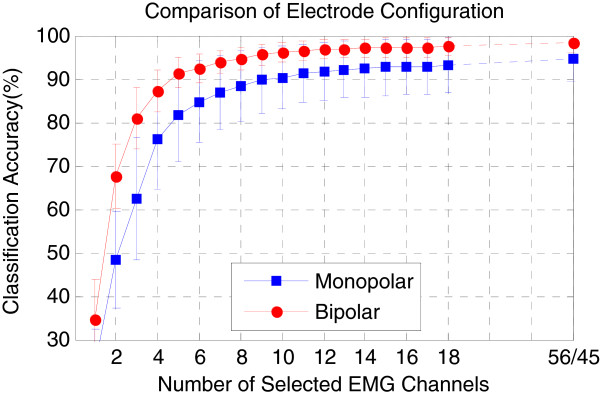
**Comparison of electrode configurations when using the EMG subset selected by MCCSP.** The TD-KNN was used as the feature-classifier combination.

Figure 7 shows the comparison of average classification accuracy across all subjects when using the EMG subsets (1-18 channels) selected by MCCSP, SFS, and FMS and their corresponding optimal feature-classifier combinations. The results indicate that applying TD-KNN to the EMG subset determined by MCCSP brought the highest classification accuracy and the best convergence when 4 to 13 optimal monopolar electrodes (Figure 7(a)) and 2 to 18 optimal bipolar electrodes (Figure 7(b)) were utilized. But the difference was not significant (p-value > 0.05).Figure 8 compares the two electrode configurations in terms of motion classification accuracy, which was obtained by using the EMG subset selected via MCCSP and the TD-KNN feature-classifier combination. The results show that the bipolar configuration was consistently better than the monopolar configuration with the increase of the number of included EMG channels. In addition, 18 EMG channels might be sufficient to get a proper motion classification performance. Because the average classification accuracy was 93.03% when using 18 optimal monopolar EMG channels, only 1.47% lower than that when using all 56 monopolar EMG channels (94.50%), and 18 optimal bipolar EMG channels brought an average classification accuracy of 95.58%, only 2.59% lower than that when using all 45 bipolar EMG channels (98.17%).

### Comparison of distribution of selected EMG channels

**Figure 9:**
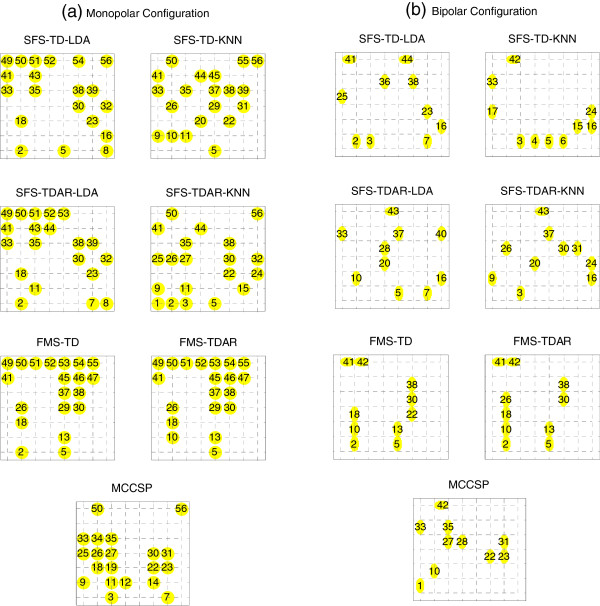
**Distribution of selected EMG channels for a subject.** Both **(a)** monopolar electrode configuration and **(b)** bipolar electrode configuration were considered.

We also analyzed the distribution of selected EMG channels when using different feature-classifier combinations for each subject. Figure 9 demonstrated a representative example from a subject (BI01), where 20 monopolar EMG channels (Figure 9(a)) and 10 bipolar EMG channels (Figure 9(b)) were selected, respectively. It was found that for both electrode configurations, the distribution of EMG channels selected by SFS varied with the choice of feature-classifier combination. When using FMS, the distribution of selected EMG channels varied slightly following the change of feature set. When using MCCSP, however, a fixed channel distribution was obtained independent of feature and classifier. The channel distributions for other subjects almost presented the similar patterns as the subject.

## Discussion

Generally speaking, using more EMG electrodes could capture more electrophysiological information that may improve the performance of movement classification in EMG-PR-based control system such as multifunctional myoelectric prostheses and active rehabilitation robots. However, this would simultaneously increase the complexity and cost of the EMG controlled systems. Thus it becomes necessary and important to find an appropriate number of electrodes and their locations for the high performance of a myoelectric control system before it is clinically viable. Theoretically, to get the optimal channels from high density channels, all the combinations of desired number of channels should be involved in the pattern recognition analysis. But it often be impractical to search a global optimal solution when a large number of channels are involved. For example, searching 6 from 56 channels would result in 32,468,436 different combinations. So some suboptimal methods have been proposed and used in the previous studies [14,16,18,19]. Two commonly used methods for channel selection are SFS and FMS, which are dependent on either classification accuracy or EMG features. While these methods might be applicable for channel selection, they have some limitations, as mentioned above in Introduction section. In this study, a novel direct channel selection algorithm was proposed and its performance in selecting the appropriate channels has been evaluated by a high-density EMG recordings from twelve mildly-impaired TBI patients. Since the newly proposed method (MCCSP) for channel selection is independent on EMG features and classification algorithms, it should be more convenient for channel selection in comparison to the SFS and FMS, especially when changing the EMG features or the classification algorithms is needed.

By examining two different types of EMG features and two pattern-recognition algorithms, we found that the TD features combining with a KNN classifier would be a better configuration for the selected EMG subset via MCCSP, and TDAR features with a LDA classifier would be a better one for the determined channels by using SFS (Figure 5). In addition, the electrode configuration is also a factor that would affect the motion classification performance. When the number of selected electrodes was increasing, the motion classification accuracy in bipolar electrode configuration reached a plateau (Figures 5 and 7) more quickly than that in monopolar electrode configuration. Furthermore, using same number of EMG channels, the bipolar electrode configuration always achieved higher classification accuracy than the monopolar electrode configuration (Figure 8). These findings are similar as the conclusions obtained by Zhou and Huang et al. [14,16]. Generally, a monopolar electrode can provide more electrophysiological information than a bipolar electrode. However, different EMG applications may need different information involved in EMG signals. For the EMG-based motion classification, the useful information should be these components of EMG signals that are characteristic of discrepancy for different movements, which could make a classifier to easily identify a movement. This may be one reason why the bipolar electrodes would outperform the monopolar electrodes in movement classification. Another possible reason underlying these findings would be that compared to a monopolar electrode, a bipolar electrode acts as a differential operator that can remove some common components such as direct-current part and common-mode noise in EMG signals. These common components in different channels might not provide any discriminate information for movement classification, even making the classification performance worse. In future study, we are interested in exploring the reasons.

As a commonly used channel selection method in EMG-PR based classification, the previous studies have shown that SFS would be an effective method in selecting the appropriate EMG channels [7,13,14,16]. However, in comparison to the proposed MCCSP, SFS was consistently worse just with one exception of using only one EMG channel (Figure 7). With SFS method, the first chosen EMG channel would be the global optimum. However, when more channels were considered, it was impossible to get the globally optimal EMG subset by using the SFS method. Compared with FMS, the proposed MCCSP performed slightly better (Figure 7) in classifying different arm/hand movements. In addition, the computational cost should be also an interesting performance metric in channel selection. In this study, we found that the average cost time taken by searching 18 optimal monopolar EMG channels was about 0.98 s when using the proposed MCCSP, significantly lower than that when using SFS (with TD feature and LDA classifier) (about 2096 s), FMS (with TD feature) (10.56 s), or FMS (with TDAR feature) (18.67 s), respectively. Additionally, a fixed combination of the selected EMG channels could be achieved when using MCCSP (Figure 9). These outcomes of this study may suggest that as an alternative, the MCCSP might be an effective and practicable choice for channel selection in the design of a practical myoelectric control system. Note that the channels selected by the three different methods in the study were inconsistent, but the similar classification performance was achieved by these selected channels. This would be because the three methods depend on different principles in channel selection. As mentioned above, as a direct channel selection algorithm the proposed MCCSP uses the raw EMG data to select the appropriate channels, the FMS and the SFS rely on EMG features and classification accuracy for searching the optimal channels, respectively. Thus the different channel combinations would be obtained by using the three methods. However, this does not mean there is no need to carefully select the channels. In the EMG-PR movement classification, it is the patterns of EMG signals from the selected channels that are considered as a whole to train and test a classifier for movement classification. Although different channel combinations were determined by the three methods, their EMG patterns all could provide identifiable information to properly classify different movements. So a similar classification performance could be achieved by the three methods. This may indicate that the channel combination for similar classification performance would be not only one.

It should be noted that all the results of this study were conducted by means of post-processing and evaluated with offline classification accuracy which might not reflect the real-time performance directly [29]. In real-time applications, the EMG signals would be easily affected by some factors like muscle contraction force [30], skin impedance [31], electrodes shift [30,32], arm position variation [33,34], and muscle fatigue [30,35]. The stability of the proposed channel selection algorithm was not considered in current study. Note that Huang et al. investigated the temporal stability of the SFS-selected electrodes by validating the EMG data recorded from follow-up experiment in terms of classification performance [16]. The repeatability of EMG signals recorded from the selected electrode sites and their inter-parameter agreement [15] would be also necessary to examine the stability of channel selection algorithm. With these important issues, we will examine the stability of the channels selected by the proposed MCCSP in our future work.

## Competing interests

The authors declare that they have no competing interests.

## Authors’ contributions

YG performed experiment design, data collection and analysis, interpretation of the results, and drafting of the manuscript, XZ was involved in interpretation of the results and the manuscript preparation, YZ and GL oversaw the study and was involved in each stage of the study, including critical revision of the manuscript. All authors read and approved the final manuscript.
